# Arthroscopic treatment of massive acromioclavicular joint ganglion cysts with color-aided visualization: a case series of 4 patients

**DOI:** 10.1016/j.xrrt.2022.06.002

**Published:** 2022-06-30

**Authors:** Yukihiro Kajita, Yusuke Iwahori, Yohei Harada, Ryosuke Takahashi, Masataka Deie

**Affiliations:** a Department of Orthopaedic Surgery, Ichinomiya Nishi Hospital, Aichi, Japan; d Department of Orthopaedic Surgery, Aichi Medical University, Aichi, Japan; b Department of Orthopaedic Surgery, Asahi Hospital, Asahi, Japan; c Department of Orthopaedic Surgery, Hiroshima University, Hiroshima, Japan

**Keywords:** Arthroscopic treatment, Acromioclavicular joint ganglion cyst, Ganglion portal, Indigo carmine

## Abstract

**Background:**

Acromioclavicular joint ganglion cysts are rare lesions that mainly arise from the degeneration of the acromioclavicular joint in elderly patients. Although surgical management may be required because of their high recurrence rate after aspiration, few reports have described arthroscopic surgical procedures to treat acromioclavicular ganglion cysts. We report the surgical results of arthroscopic ganglionectomy with color-aided visualization for massive acromioclavicular ganglion joint cysts.

**Methods:**

This retrospective case series examined patients identified with massive ganglion cysts that were localized above the acromioclavicular joint. All patients underwent an arthroscopic removal of subacromial synovium and subsequent injection of indigo carmine into the ganglion. The distal end of the clavicle was excised arthroscopically from the inferior surface, and the ganglion stalk was confirmed using indigo carmine for enhanced visualization and magnification. A ganglion portal was created, and the ganglion cyst was resected with the aid of the dye.

**Results:**

Four female patients, aged 78-90 years, were identified with a massive acromioclavicular joint ganglion cyst. Plain radiography showed joint degeneration in the acromioclavicular joint, and magnetic resonance imaging scans showed fluid-filled mass formation. Although all patients initially underwent multiple aspirations of the ganglion cyst, we opted for surgical intervention because of its persistent recurrence. Three patients exhibited concurrent rotator cuff tears, and one patient had a prior history of cuff repair with no retear. After arthroscopic ganglionectomy with color-aided visualization for massive acromioclavicular ganglion joint cysts, none of the patients have shown recurrences at 2 years postoperatively.

**Conclusion:**

Novel aspects of this case series include the use of indigo carmine to provide a better visualization and identification of the ganglion stalk under arthroscopy. Furthermore, a ganglion portal is useful for achieving complete resection of the indigo carmine–stained ganglion cyst. Color-aided visualization using indigo carmine and the construction of a ganglion portal were useful techniques for performing arthroscopic ganglionectomy in patients with a massive acromioclavicular joint ganglion cyst.

Acromioclavicular (AC) joint ganglion cysts are rare lesions that mainly arise from the degeneration of the AC joint in elderly patients. The first cases of AC joint ganglion cysts associated with rotator cuff tears were presented by Craig in 1986.^3^ The ganglion cyst forms by glenohumeral joint fluid leakage through the full-thickness rotator cuff tear into the subacromial bursa and into a degenerative AC joint, eventually causing distension of the superior AC joint capsule. For fluid to pass into the AC joint, the inferior capsule must be torn. Increased synovial fluid production and the subsequent formation of a check valve mechanism can cause the synovial fluid to escape into the AC joint capsule, thereby creating the ganglion cyst. AC joint ganglion cysts may induce AC joint pain as well as cosmetic concerns.

Treatment options of AC joint ganglion cysts include observation, aspiration, and surgical excision. Aspiration and simple ganglion cyst excision often fail because the underlying problem of the rotator cuff and AC joint is not corrected.^4^^,^^9^ If the rotator cuff tear is irreparable, the AC joint should be excised to remove the check valve effect. Some authors recommend AC joint excision.^6^^,^^7^^,^^14^^,^^15^^,^^17^, ^18^, ^19^, ^20^^,^^26^, ^27^, ^28^, ^29^, ^30^, ^31^ Most studies have recommended open excision,^5^, ^6^, ^7^^,^^14^^,^^15^^,^^17^, ^18^, ^19^, ^20^^,^^26^, ^27^, ^28^, ^29^ and reports on arthroscopic surgery are scarce in the literature. Because patients with AC joint ganglion cysts are often elderly and thus more vulnerable to perioperative trauma, we performed a new arthroscopic technique to minimize surgical invasiveness. We report the surgical results of arthroscopic ganglionectomy with color-aided visualization for massive AC ganglion joint cysts.

## Methods

We retrospectively reviewed our database of surgical shoulder procedures that were performed at our hospital from 2012 to 2018. Patients who underwent arthroscopic ganglionectomy with over 2-year follow-up were identified. Patients were diagnosed as having AC joint ganglion cysts using magnetic resonance imaging (MRI). Although conservative therapy was initially performed after diagnosis, all patients underwent surgery due to persistent recurrences after at least 5 aspirations that were carried out at the outpatient clinic.

This study complies with the Declaration of Helsinki and was performed according to the Institutional Review Board guidelines of Aichi Medical University (Approval Number 2021-H131).

### Surgical technique

**Figure 1:**
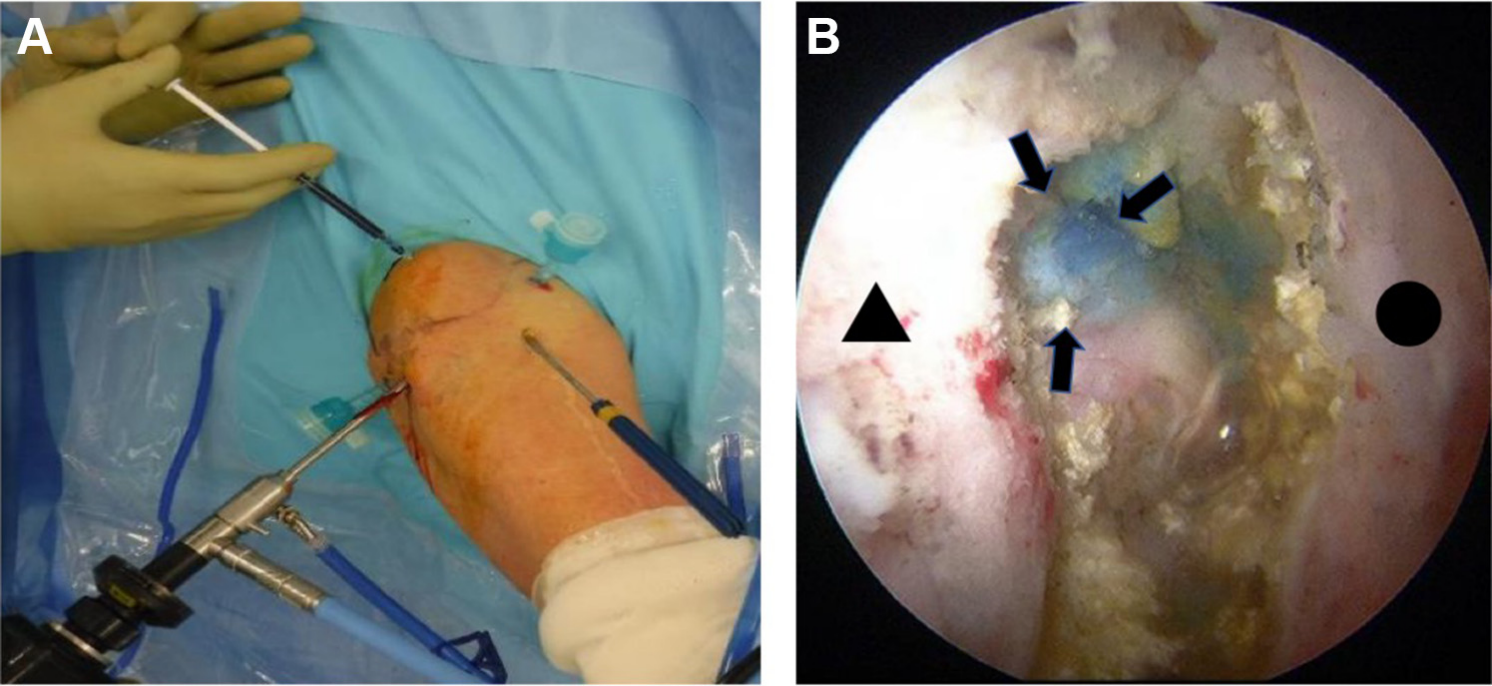
(**A**) Indigo carmine injection into the ganglion cyst. (**B**) The inferior surface of the AC joint was observed from the posterior portal using 70-degree arthroscope. The distal end of the clavicle was removed using a shaver, and the stalk of the indigo carmine–stained ganglion was identified. •, medial acromion; ▲, distal end of clavicle; , ganglion stalk. *AC*, acromioclavicular.

**Figure 2:**
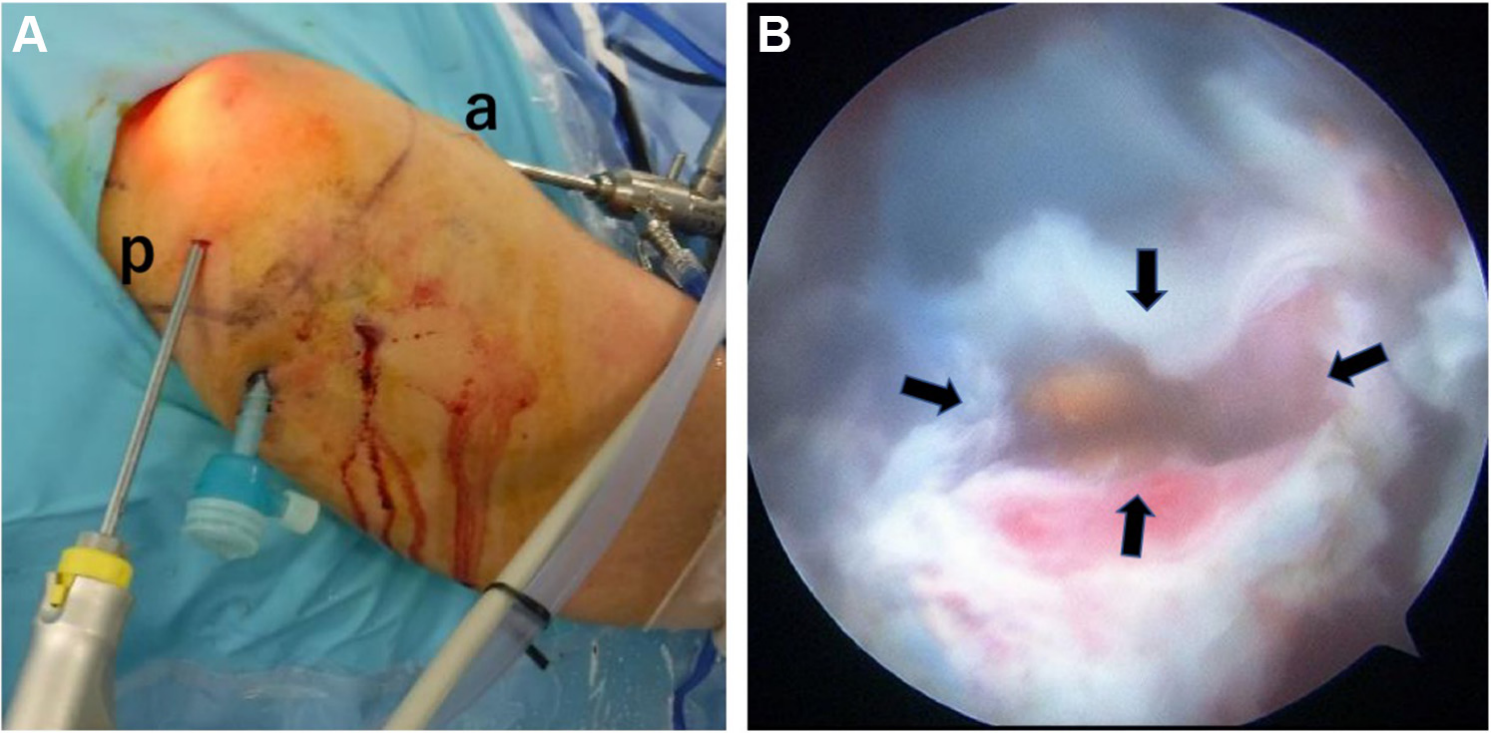
(**A**) Creation of a ganglion portal anterior and posterior to the ganglion (*a*. anterior portal; *p*, posterior portal). (**B**) Indigo carmine–stained ganglion cyst (, ganglion stalk). A shaver was inserted from the anterior portal, and the indigo carmine–stained ganglion cyst was excised.

Surgeries were performed under general anesthesia with additional interscalene nerve block. Patients were positioned in the beach chair position. Arthroscopy was performed using standard posterior and anterior portals. The subacromial synovium was first excised, and 2-3 mL of indigo carmine was administered by intralesional injection into the ganglion using a 22-gauge needle (Fig. 1*A*). Then, the AC joint was observed under arthroscopy from the inferior surface, and the distal end of the clavicle was excised using a shaver until the stalk of the indigo carmine–stained ganglion could be identified (Fig. 1*B*). The stained ganglion stalk was magnified under arthroscopy, and the check valve was subsequently destroyed. Next, 2 ganglion portals were created at a site 5 cm anterior and posterior to the ganglion (Fig. 2*A*). The inside of the ganglion was observed from the ganglion portal, and the surgery was complete after the indigo carmine–stained ganglion cyst was excised using a shaver (Fig. 2*B*). The shaver was carefully swept back and forth to débride the cyst capsule so as not to damage the surrounding soft tissues that were not stained.

## Results

Four female patients, aged 78-90 years, were identified with a massive AC joint ganglion cyst. Plain radiography showed joint degeneration in the AC joint, and MRI scans showed fluid-filled mass formation. Three patients exhibited concurrent rotator cuff tears, and one patient had a prior history of cuff repair with no retear. Although all 4 patients complained of AC joint pain, their shoulder functions were relatively preserved, and they only requested the removal of their massive AC joint ganglion cysts. No recurrence has been observed after arthroscopic ganglionectomy. The details of individual cases are described in the following sections.

### Case 1

**Figure 3:**
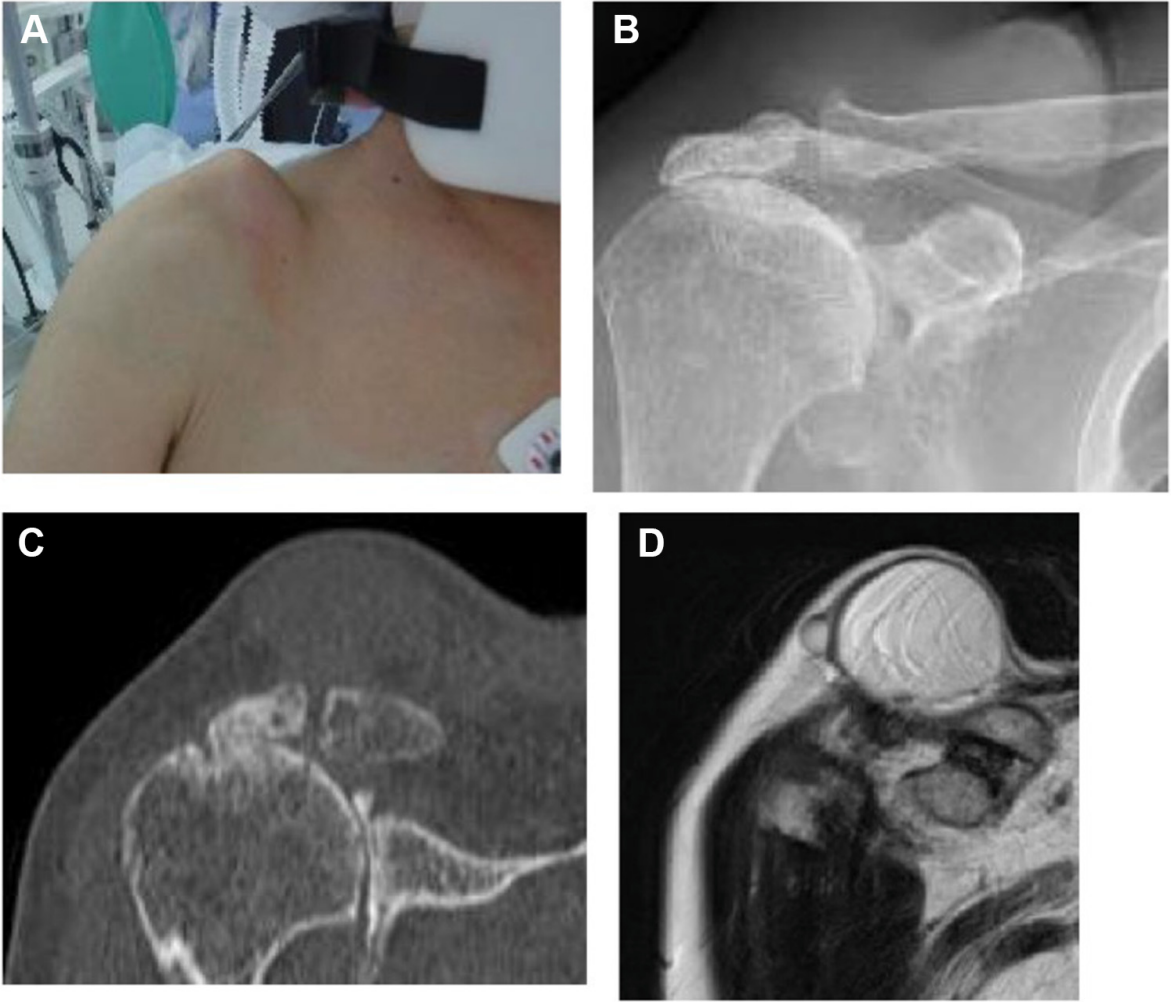
Preoperative swelling of the acromioclavicular joint and preoperative images of Case 1. (**A**) There is notable swelling of the acromioclavicular joint. (**B, C**) the humeral head is translated superiorly, and the acromioclavicular joint shows degeneration. (**D**) MRI scan shows a cyst in the superior part of the acromioclavicular joint. *MRI*, magnetic resonance imaging.

**Figure 4:**
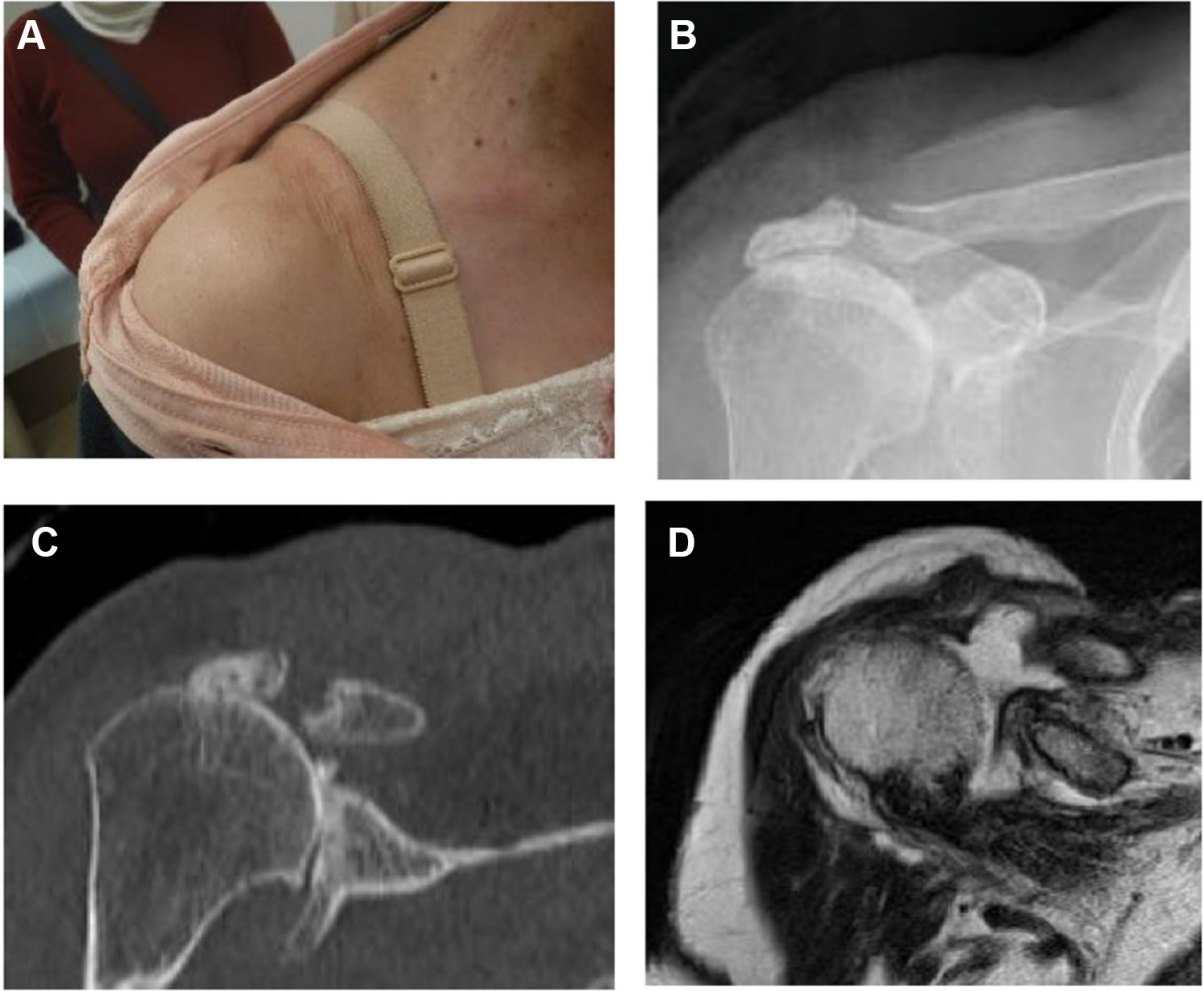
Postoperative swelling of the acromioclavicular joint and postoperative images of Case 1. (**A**) The swelling of the acromioclavicular joint has disappeared. (**B, C**) Approximately 3 mm is resected from the distal end of the clavicle. (**D**) MRI scan shows that the cyst in the superior section of the acromioclavicular joint has disappeared. *MRI*, magnetic resonance imaging.

A 79-year-old female complained of pain in the right AC joint 6 months before examination with swelling on the superior side of the AC joint. She was diagnosed with an AC joint ganglion cyst at another hospital. She was referred to our hospital for surgery, as her ganglion cyst persistently recurred after multiple ultrasound-guided aspirations. Radiographs revealed an acromial spur, degenerative changes of AC joint, early glenohumeral degenerative changes, and a large soft-tissue mass. MRI showed a full-thickness rotator cuff tear involving the supraspinatus, infraspinatus, and subscapularis in addition to a massive cyst (50 mm × 55 mm) over the AC joint (Fig. 3). As the patient was able to maintain shoulder joint function but experienced mild pain around the AC joint and shoulder, arthroscopic surgery was performed. At 2-year postoperative follow-up, no recurrence of AC joint ganglion cyst was observed, the shoulder joint function was maintained, and mild pain remained around the AC joint (Fig. 4).

### Case 2

**Figure 5:**
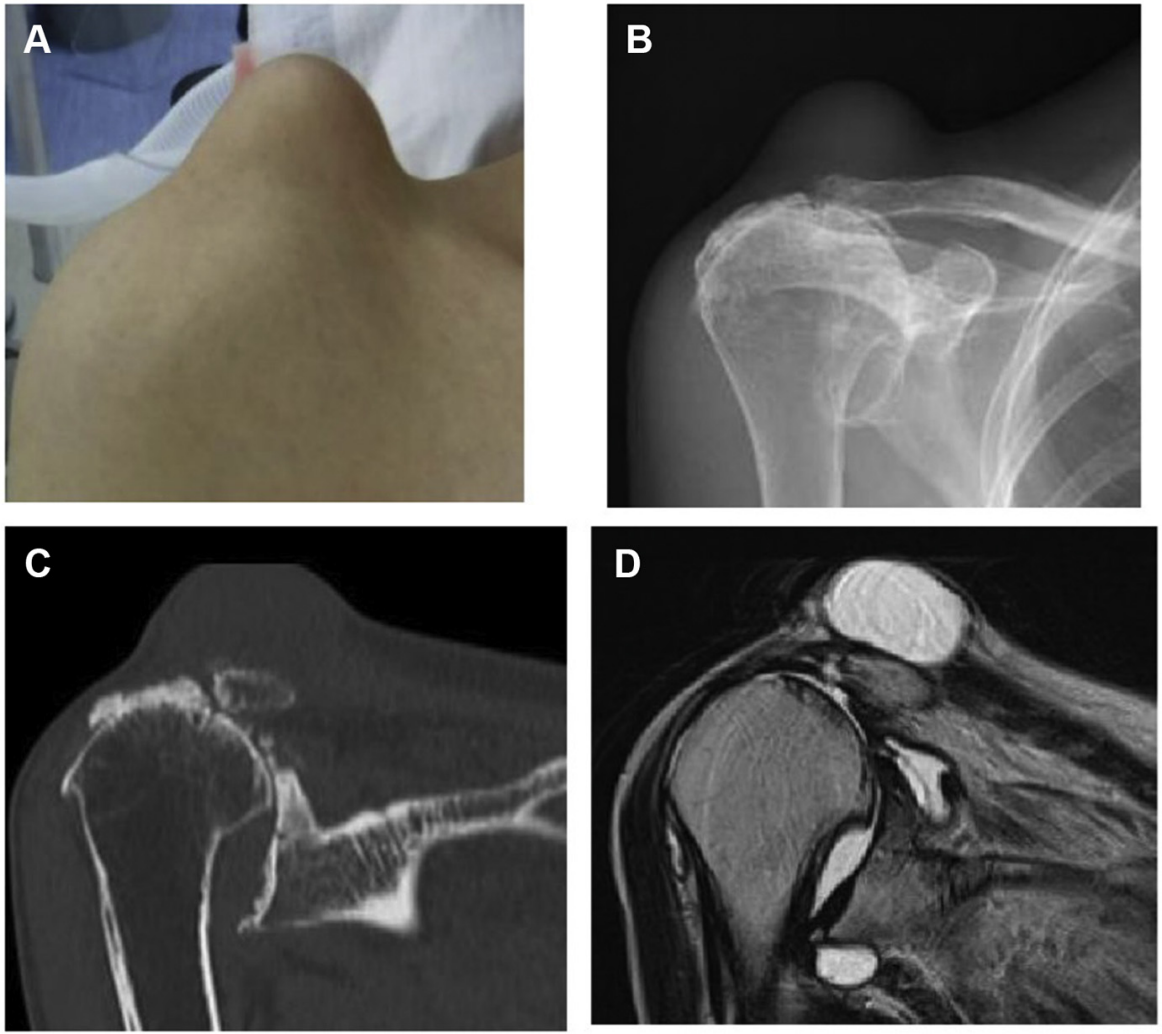
Preoperative swelling of the acromioclavicular joint and preoperative images of Case 2. (**A**) There is notable swelling of the acromioclavicular joint. (**B, C**) The humeral head is translated superiorly, and the acromioclavicular joint shows degeneration. (**D**) MRI scan shows a cyst in the superior part of the acromioclavicular joint. *MRI*, magnetic resonance imaging.

**Figure 6:**
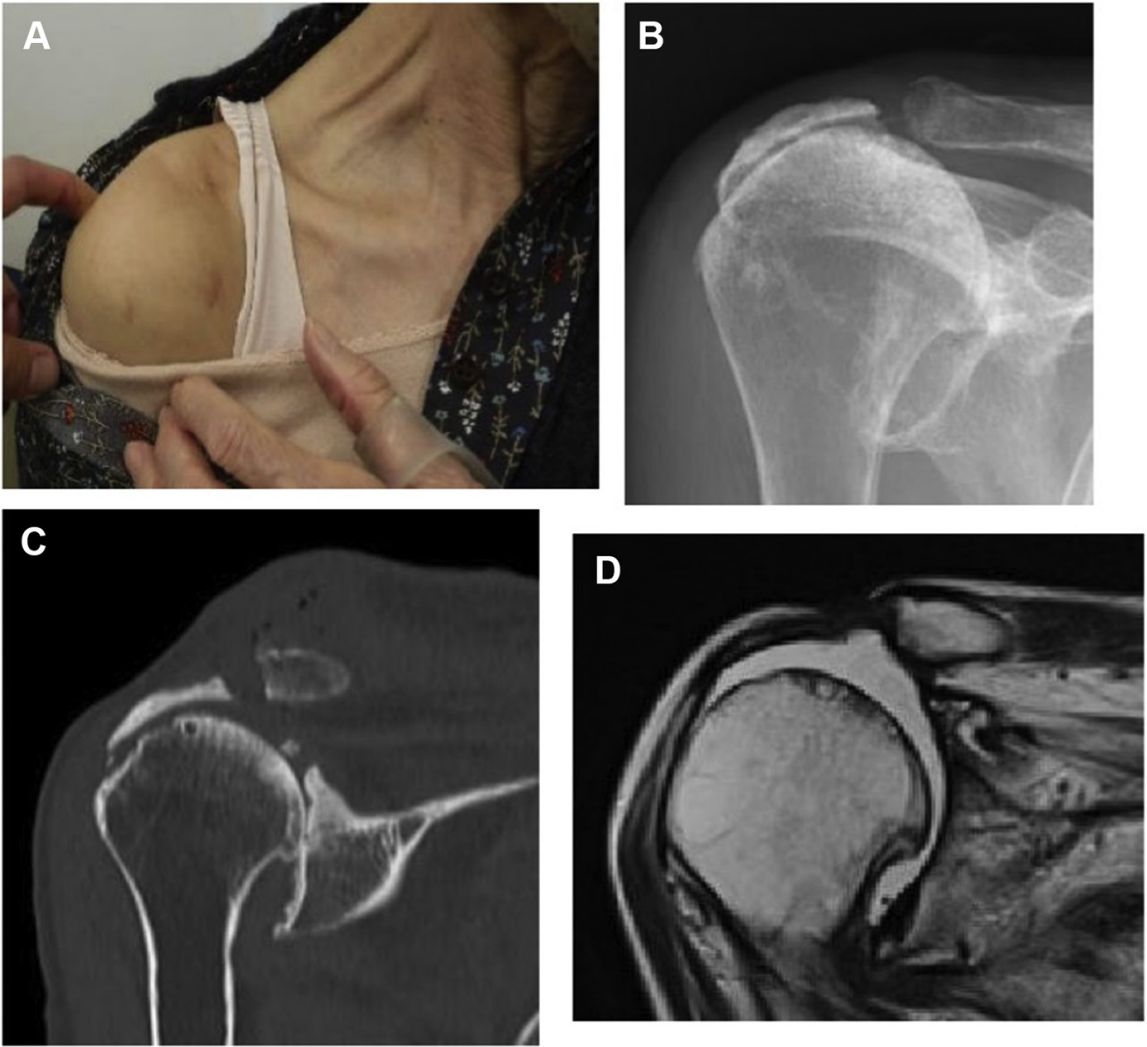
Postoperative swelling of the acromioclavicular joint and postoperative images of Case 2. (**A**) The swelling of the acromioclavicular joint has disappeared. (**B, C**) Approximately 3 mm is resected from the distal end of the clavicle. (**D**) MRI scan shows that the cyst in the superior section of the acromioclavicular joint has disappeared. *MRI*, magnetic resonance imaging.

A 78-year-old female complained of mild pain in her right shoulder for several years, but she did not visit a hospital as the pain did not interfere with her activities of daily living. She experienced swelling in the superior part of her right AC joint for a year, and she visited another hospital for examination. She underwent multiple aspirations but experienced persistent recurrences of the condition. She requested surgery due to cosmetic concerns and was referred to our hospital. Plain radiography and computed tomography scans showed Type IIA degenerative changes (Hamada classification system) to her shoulder joint and AC joint, and the MRI scan showed a massive rotator cuff tear in addition to a massive cyst (43 mm × 54 mm) over the AC joint (Fig. 5). Arthroscopic surgery was performed, and no recurrence was observed at 3 years postoperatively (Fig. 6).

### Case 3

**Figure 7:**
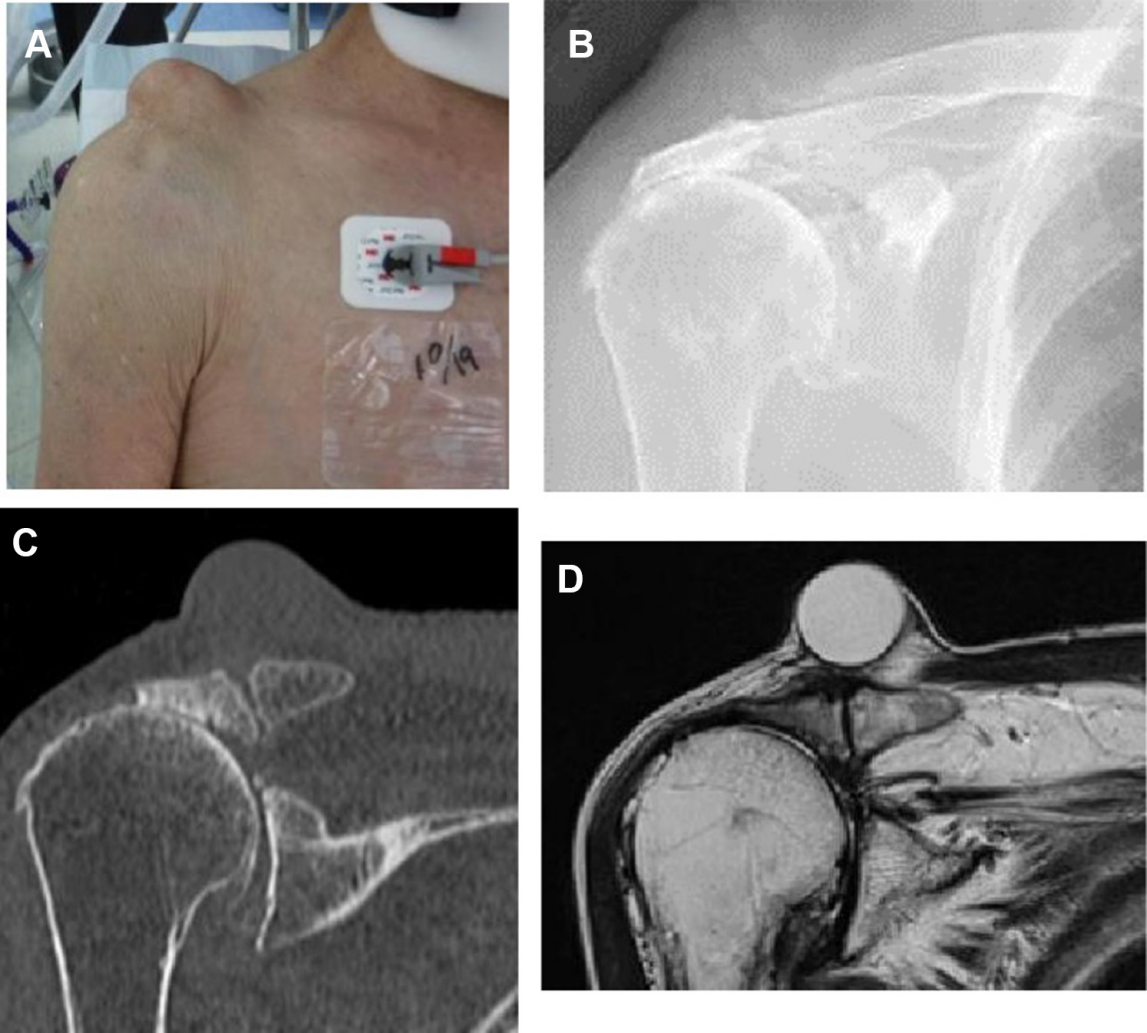
Preoperative swelling of the acromioclavicular joint and preoperative images of Case 3. (**A**) There is notable swelling of the acromioclavicular joint. (**B, C**) The humeral head is translated superiorly and the acromioclavicular joint shows degeneration. (**D**) MRI scan shows a cyst in the superior part of the acromioclavicular joint. *MRI*, magnetic resonance imaging.

**Figure 8:**
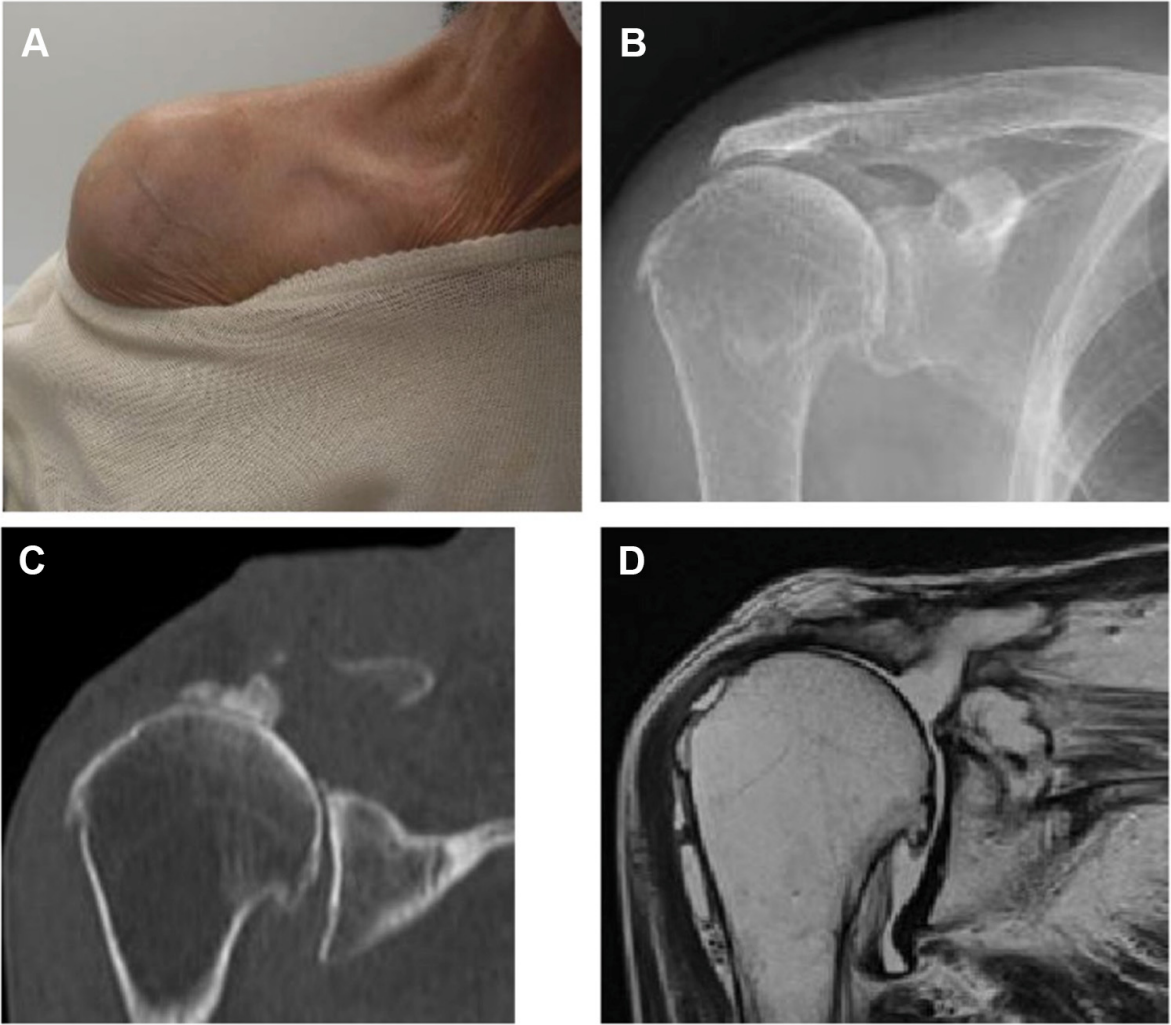
Postoperative swelling of the acromioclavicular joint and postoperative images of Case 3. (**A**) The swelling of the acromioclavicular joint has disappeared. (**B, C**) approximately 3 mm is resected from the distal end of the clavicle. (**D**) MRI scan shows that the cyst in the superior section of the acromioclavicular joint has disappeared. *MRI*, magnetic resonance imaging.

A 90-year-old female had mild pain in her right shoulder for the past 20 years and gradually found difficulty in raising her shoulder. She underwent aspiration of her AC ganglion joint cyst once per week for 6 months at another hospital; however, she wanted to undergo a curative treatment and was referred to our hospital. The active abduction of her right shoulder joint was 120 degrees, and her active external rotation was about 30 degrees. Plain radiography and computed tomography scans showed Type IIA degenerative changes (Hamada classification system) to her shoulder joint and AC joint, and the MRI scan showed a massive rotator cuff tear in addition to a massive cyst (42 mm × 40 mm) over the AC joint (Fig. 7). We opted to perform arthroscopic surgery due to the patient’s old age and low demand. She completed the surgery without complications, and the pain around the AC joint had completely subsided. She had no recurrence at 2 years postoperatively (Fig. 8).

### Case 4

**Figure 9:**
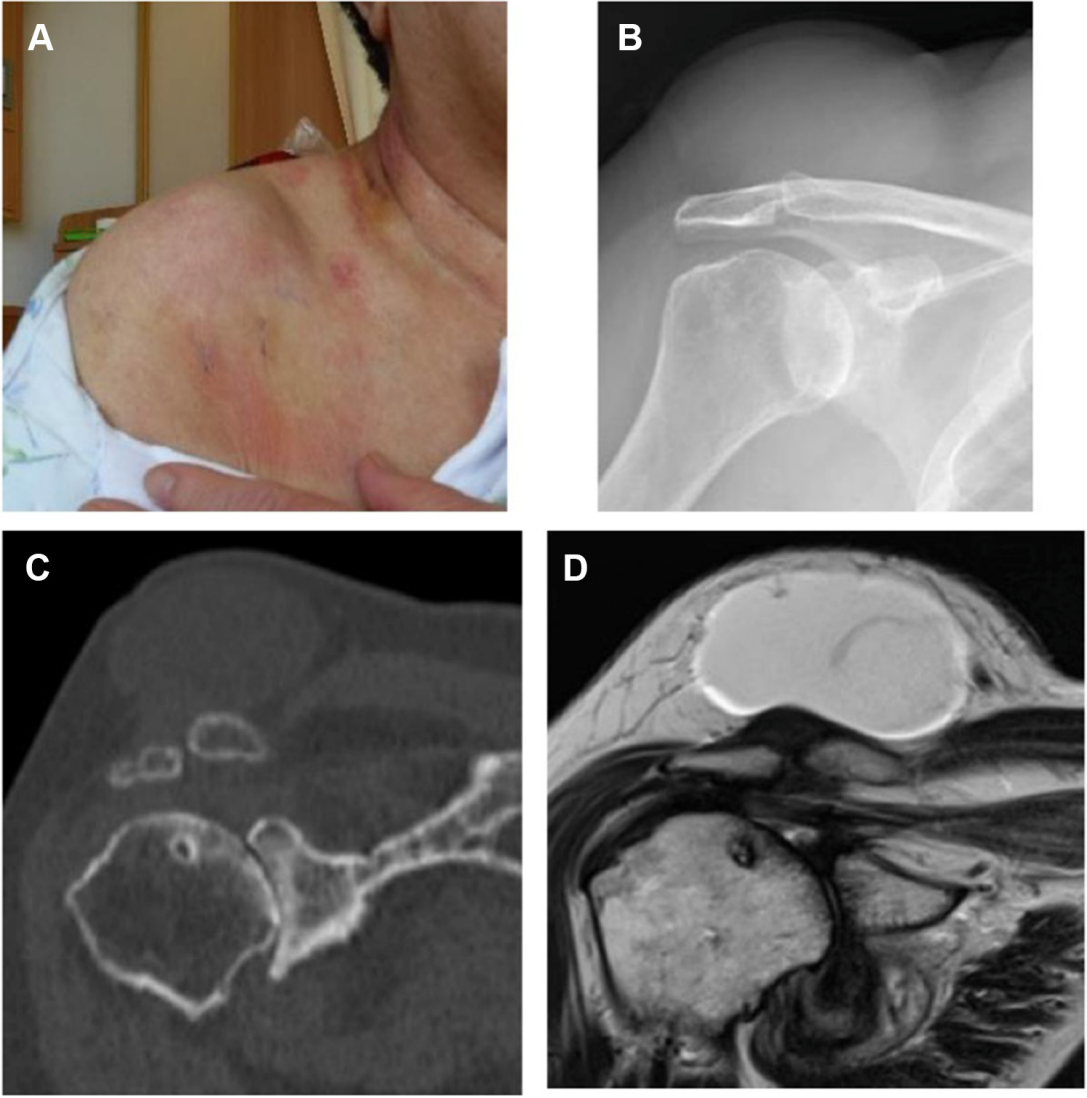
Preoperative swelling of the acromioclavicular joint and preoperative images of Case 4. (**A**) There is notable swelling of the acromioclavicular joint. (**B, C**) The acromioclavicular joint shows degeneration. (**D**) MRI scan shows a cyst in the superior part of the acromioclavicular joint. No retear is observed after cuff repair. *MRI*, magnetic resonance imaging.

**Figure 10:**
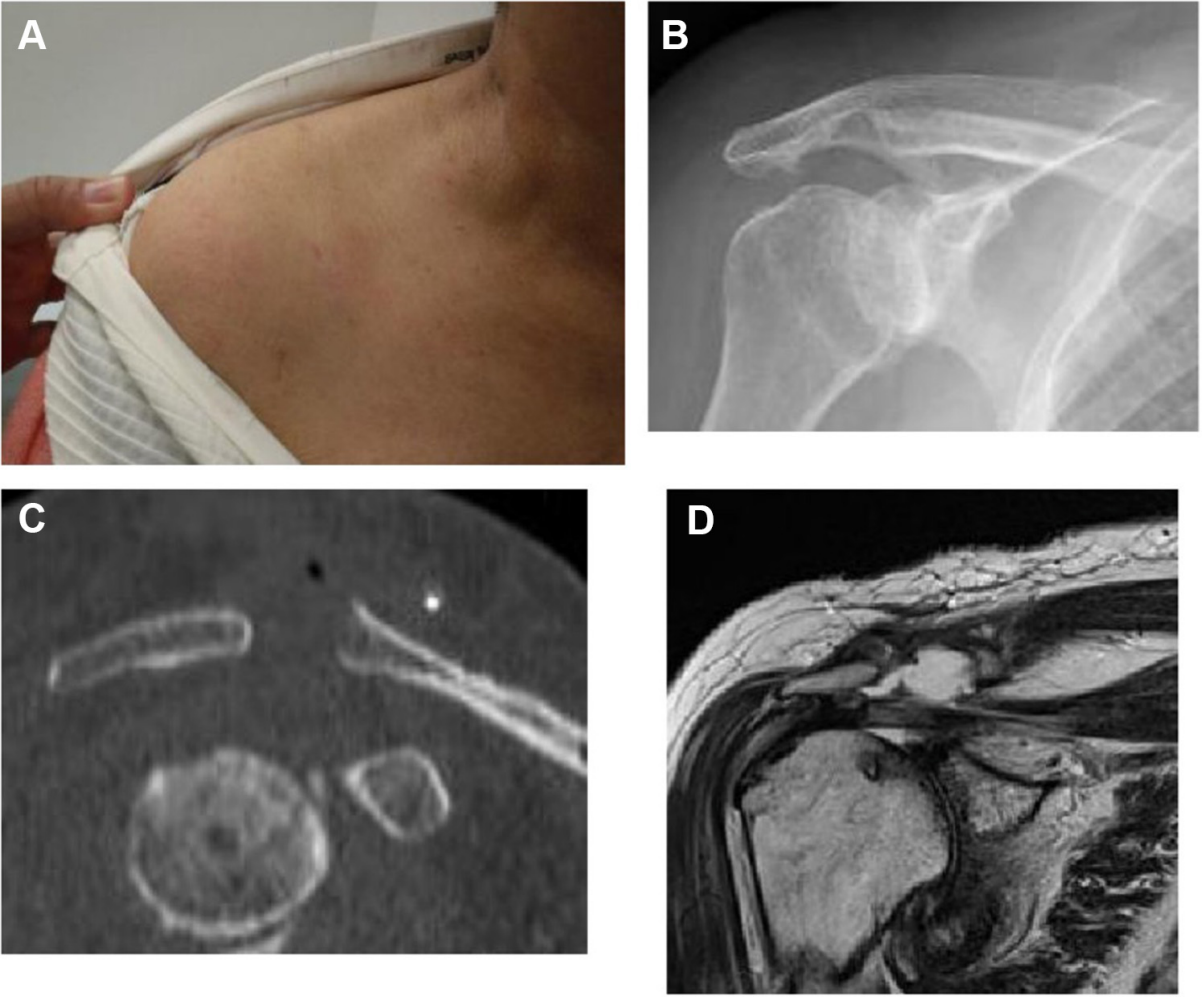
Postoperative swelling of the acromioclavicular joint and postoperative images of Case 4. (**A**) The swelling of the acromioclavicular joint has disappeared. (**B, C**) Approximately 5 mm is resected from the distal end of the clavicle. (**D**) MRI scan shows that the cyst in the superior section of the acromioclavicular joint has disappeared. *MRI*, magnetic resonance imaging.

A 73-year-old female underwent a rotator cuff repair on her right shoulder when she was 68 years of age. Although her shoulder joint function had improved after the operation, she noticed a swelling above her right AC joint 1 year prior. The patient underwent multiple aspirations but chose to undergo surgery due to the persistent recurrences of pain around the AC joint. She had a massive cyst (65 mm × 80 mm) in the superior part of the AC joint on MR. No retear of the rotator cuff was observed (Fig. 9). She underwent arthroscopic surgery and was followed up for 2 years after surgery. No recurrence was observed, and the pain around the AC joint had completely subsided (Fig. 10).

## Discussion

AC joint ganglion cyst is a rare sequelae of advanced AC joint arthritis. They are classified as Type 1 and Type 2 ganglion cysts in accordance with its etiology. Type 1 ganglion cysts are seen in advanced AC joint arthritis, wherein the formation of synovial inflammation and cysts are limited to the joint and are not associated with rotator cuff tear.^3^^,^^5^^,^^6^^,^^11^^,^^25^ On the other hand, the pathology of the Type 2 ganglion cyst is dependent on rotator cuff morphology.^6^^,^^25^^,^^29^ Complete tear of the rotator cuff predisposes the patient to superior migration of the humeral head, leading to irritation and deterioration of AC joint capsule. Increased synovial fluid production and formation of check valve cause the escape of the synovial fluid into the AC joint capsule, creating the ganglion cyst.

In this study, a rotator cuff tear with Type 2 ganglion cyst was found on preoperative MRI scans of Cases 1, 2, and 3. Although Case 4 had a prior history of rotator cuff repair, the ganglion cyst was classified as Type 1, as there was no history of rehear.

The treatment of AC joint ganglion cysts can be either conservative or surgical, depending on various factors such as symptoms, patient age, and shoulder function. Conservative treatment is recommended for elderly patients with few or no symptoms and good shoulder function. Conversely, patients with chronic pain and swelling around the AC joint should be addressed with surgical treatment. Previous reports have described an open resection of the distal clavicle, including the AC joint ganglion cyst and check valve, total shoulder arthroplasty, hemiarthroplasty, and reverse total shoulder arthroplasty.^1^, ^2^, ^3^, ^4^, ^5^, ^6^^,^^8^^,^^14^^,^^15^^,^^17^, ^18^, ^19^, ^20^^,^^22^^,^^25^, ^26^, ^27^, ^28^, ^29^, ^30^, ^31^ In this case series, we opted to perform arthroscopic surgery. To our knowledge, only one report by Mullet et al has described an arthroscopic procedure for treating AC joint ganglion cysts.^19^ The advantages of arthroscopic treatment are that it is less invasive and allows excellent visualization of the glenohumeral joint, subacromial bursa, and the AC joint ganglion cyst. One report has described the need for destroying the check valve in addition to repairing the rotator cuff to treat Type 2 AC joint ganglion cysts.^27^ On the other hand, there are also reports that suggest there is no need to perform cuff repair even in those with an irreparable rotator cuff tear and that the resection of the distal end of the clavicle and the destruction of the check valve is sufficient for treating the ganglion cysts.^10^^,^^25^, ^26^, ^27^ In our series, there were 3 cases of Type 2 cysts. Rotator cuff repair was deemed difficult because of the torn rotator cuff that had already regressed and the superiorization of humeral head. However, the active range of motion of the shoulder joint was maintained to the extent that it did not interfere with activities of daily living; therefore, we did not perform rotator cuff repairs or arthroplasty. In our case, postoperative rotator cuff repair was not necessary for preventing the recurrence of AC joint ganglion cysts, considering the AC joint ganglion cysts were believed to be the cause of the check valve.^4^^,^^9^

We injected indigo carmine into the ganglion to arthroscopically identify the ganglion stalk and cyst. Indigo carmine enables an easier visualization of ganglion stalks and cysts under arthroscopic view. Indigo carmine is generally believed to be a safe, biologically inert substance, and is often used during cystoscopy when evaluating for lower urinary tract integrity after gynecologic surgery.^7^^,^^21^ In the field of orthopedic surgery, there have been reports of its use in Baker cysts and ganglia of the hand and foot.^23^^,^^24^^,^^32^ To our knowledge, this is the first report of surgery using indigo carmine for AC joint ganglion cysts. It has been reported that indigo carmine can potentially cause hypotension and bradycardia when intravenously administered.^7^^,^^21^ However, when injecting indigo carmine into an AC joint ganglion cyst, the dye is injected directly above the AC joint ganglion cyst and does not enter the blood vessel; thus, the dye can be used safely and effectively.

In the surgical procedure described in this study, we created 2 ganglion portals near the AC joint ganglion cyst when the ganglion cyst was arthroscopically resected. Although there are several reports that describe the use of ganglion portals for ganglion cysts that occur near the wrist and elbow joints,^12^^,^^13^^,^^16^ only one report by Mullet et al^19^ has described the use for AC joint ganglion cysts. Although neurovascular damage should be avoided when creating a ganglion portal in the wrist and elbow joints, the arthroscopic construction of a ganglion portal is relatively safe around the AC joint, as there are no major neurovascular structures surrounding the AC joint. In this study, ganglion portals were created after injecting indigo carmine into the ganglion cyst. By observing the inside of the ganglion cyst from the ganglion portal, the cyst stained with indigo carmine improved its visualization, enabling a complete resection of the cyst. The portal proved to be a useful aid in performing arthroscopic ganglionectomy.

## Conclusion

Arthroscopic ganglionectomy was performed on massive AC joint ganglion cysts in 4 cases, and there was no recurrence at 2 years postoperatively. Injecting indigo carmine into the ganglion cyst and excision of the ganglion cyst using the ganglion portal were effective methods for arthroscopic surgery of the massive AC joint ganglion cyst.

## Disclaimers:

Funding: No funding was disclosed by the authors.

Conflicts of interest: The authors, their immediate families, and any research foundation with which they are affiliated have not received any financial payments or other benefits from any commercial entity related to the subject of this article.
